# Long-term strength and balance training in prevention of decline in muscle strength and mobility in older adults

**DOI:** 10.1007/s40520-019-01155-0

**Published:** 2019-03-04

**Authors:** Eeva Aartolahti, Eija Lönnroos, Sirpa Hartikainen, Arja Häkkinen

**Affiliations:** 1 Faculty of Sport and Health Sciences, University of Jyvaskyla, Jyvaskyla, Finland; 2 Institute of Public Health and Clinical Nutrition, Department of Geriatrics, University of Eastern Finland, Kuopio, Finland; 3 School of Pharmacy, University of Eastern Finland, Kuopio, Finland; 4 Kuopio Research Centre of Geriatric Care, University of Eastern Finland, Kuopio, Finland; 5 Department of Physical and Rehabilitation Medicine, Central Finland Health Care District, Jyvaskyla, Finland

**Keywords:** Balance, Exercise, Geriatric assessment, Intervention, Physical performance, Resistance training

## Abstract

**Background:**

Reductions in muscle strength and poor balance may lead to mobility limitations in older age.

**Aims:**

We assessed the effects of long-term once-weekly strength and balance training (SBT) on muscle strength and physical functioning in a community-based sample of older adults.

**Methods:**

182 individuals [130 women and 52 men, mean age 80 (SD ± 3.9) years] underwent supervised SBT as part of the Geriatric Multidisciplinary Strategy for the Good Care of the Elderly study. Training was offered once a week for 2.3 years. Isometric knee extension and flexion strength, chair rise, maximal walking speed, timed up and go (TUG) and Berg Balance Scale (BBS) were measured at baseline, after 2-year training and at post intervention follow-up. A linear mixed model was used to examine the change in physical functioning over time.

**Results:**

During the intervention, both women (2.5 s, *p* < 0.001) and men (1.4 s, *p* = 0.013) improved their chair rise capacity. Women’s knee extension and flexion strength improved by 14.1 *N* (*p* = 0.003) and 16.3 *N* (*p* < 0.001), respectively. Their maximal walking speed also improved by 0.08 m/s (*p* < 0.001). In men, no changes in muscle strength or walking speed occurred during training or follow-up. No changes in BBS and TUG were observed at the end of the intervention, but decrease in BBS was observed at post-intervention follow-up in men.

**Conclusions:**

In community-dwelling older adults with variety in health and functioning supervised strength and balance training once a week may help to prevent age-related decline in mobility and muscle strength.

## Introduction

In old age, independent and safe mobility is an important factor for maintaining one’s quality of life and independence [1]. Especially reductions in muscle strength and poor balance may lead to mobility limitations and further disability [2]. The loss in muscle strength may be attributed to the aging process, disuse of muscles, disease or malnutrition and longitudinal studies have shown that the rate of lower limb strength lost is approximately 2.5–4% per year after 75 years of age [3]. The combined effect of muscle and balance impairment increases the risk of walking disability compared to having only one or other of these impairments [4, 5]. While decreased physical activity may accelerate sarcopenia-related factors such as the loss of muscle mass and strength, has physical activity and exercise shown to promote healthy aging and prevent mobility limitations [6]. Thus strength and balance training (SBT) is an important component of physical activity guidelines for older adults and is also recommended for the prevention of falls and disability [7].

The effects of SBT on physical functioning and disability have been studied in several randomized controlled trials [8–12]. While the strength training as an isolated intervention has not been shown to be uniformly effective in improving balance performance [13], the functional balance exercise in addition to machine-driven resistance training has shown to improve functional performance compared to strength training only [14]. The majority of previous studies are based on short-term interventions among either healthy older adults or among older adults undergoing rehabilitation for specific clinical conditions [10, 12]. More recently, the evidence on the effectiveness of SBT on community-dwelling frail population has been strengthening [15, 16]. Nonetheless, among the long-term interventions, the intervention length only seldom has exceeded 1 year [9]. When aiming to increase physical activity and health at the population level, studies with extended follow-up periods and on a larger number of individuals with a wide variety of functional levels are needed.

Even less than 10% of the population aged 75 years or over participate in strength training at the level recommended in physical activity guidelines [17]. Therefore, even once-weekly training would represent a substantial increase in physical activity among frailest population. In addition, once-weekly training could reasonably be expected to help in the prevention of age-related loss of muscle strength [18, 19]. Taking into account the limited resources and the growing percent of older adults in societies, it is important to seek optimal time and cost effectiveness also in physical activity programmes.

The purpose of this prospective study was to assess the effects of the once-weekly, supervised, over 2-year SBT on the muscle strength and physical functioning in a community-based sample of people aged 75 years and older. In addition, maintenance of the intervention effects was evaluated at post-intervention follow-up. We hypothesized that SBT offered once-weekly would prevent the decline of muscle strength and physical functioning and that effects would start to decrease after the intervention concluded.

## Methods

### Subjects

This study is part of a larger GeMS-project (Geriatric Multidisciplinary Strategy for the Good Care of the Elderly), where 1000 people aged 75 years and older who lived in Kuopio, Finland, in November 2003 were invited to the study [20]. The enrollment took place from January 2004 to September 2004. The 339 community-dwelling individuals of the intervention group received physical activity counseling annually and were offered an opportunity to participate in SBT once a week at the gym. Of those, 182 started the SBT program and were included in the analyses. Eligibility for the SBT was based on clinical examination by a study physician. The training could be commenced later if the participant had permanent or transient contraindications for training, such as an unstable acute or chronic medical condition or was recovering from an operation. An inclusion criterion was the ability to move independently or with minimal help in the gym. The gyms were also accessible for participants with assistive devices. Those participants who did not start training (*n* = 157) were older and had lower health and physical functioning levels compared with SBT-initiators (*n* = 182) [21]. The mean age of the participants at baseline was 79.7 (SD ± 3.9, range 75–98) years, and 71% of them were women (Table 1). Women had lower grip strength (*p* < 0.001), higher independence in instrumental activities of daily living (*p* < 0.001) and they more often used a walking aid (*p* = 0.046) compared to men.

**Table 1:** Baseline descriptive characteristics of participants

	All *n* = 182	Men *n* = 52	Women *n* = 130
Demographics			
Age, (years)	79.7 ± 3.9	79.1 ± 3.8	80.0 ± 4.0
Education, (years)	6 [5, 10]	7 [6, 10]	6 [5, 9]
Health status			
Functional comorbidity index	2.1 ± 1.5	2.1 ± 1.5	2.1 ± 1.5
MMSE	27.7 ± 2.5	27.4 ± 3.2	27.8 ± 2.2
Self-rated health, *N* (%)			
Poor/very poor	15 (8)	4 (8)	11 (8)
Moderate	88 (48)	26 (50)	62 (48)
Good/very good	79 (43)	22 (42)	57 (44)
Physical functioning			
IADL	7.2 ± 1.4	6.4 ± 1.9	7.5 ± 0.9
Grip strength, (kg)	25.4 ± 9.4	35.4 ± 9.9	21.4 ± 5.3
Physical activity, *N* (%)			
Low	51 (28)	11 (21)	40 (31)
Moderate	93 (51)	26 (50)	67 (51)
High	38 (21)	15 (29)	23 (18)
Use a walking aid, *N* (%)	39 (21)	6 (12)	33 (26)

Data are presented as mean ± SD, N (%) or median [IQR]

Ten percent (*n* = 17) of the SBT-participants were lost by the 3-year follow-up point due to death, poor health or refusal to participate. In addition, there were missing results for ten participants in knee extension and flexion strength tests and for 17 participants in chair rise test at the 2 years measurement. The GeMS study was approved by the Research Ethics Committee of Northern Savo Hospital District and Kuopio University Hospital. Written informed consent was obtained from all individual participants included in the study.

### Measures

Three trained nurses, two physiotherapists and two physicians collected the comprehensive geriatric assessment data. Socio-demographic factors, health status, cognitive and physical functioning and the ability to perform instrumental activities of daily living were assessed. The assessments were repeated annually.

Unilateral maximal isometric knee extension and flexion strength with a knee angle of 60° was measured in a sitting position using an adjustable dynamometer chair (Good Strength; METITUR OY, Finland). Participants were allowed three maximal efforts for each leg, and the best performance with the highest value was accepted as the result. There was 1-min rest interval between each attempt. Grip strength was measured in the seated position with the elbow flexed 90° using a dynamometer (Saehan Corporation, South Korea). One maximal effort for both hands was allowed, and the result from the stronger hand was used in the analyses. Isometric contraction lasting approximately 3 s was used in all strength measurements.

A modified chair stand test [22] was used to assess the ability to perform sit-to-stand and stand-to-sit tasks five times as fast as possible. As a modification of the original test, hands were held at their sides, and participants were allowed to use their hands for assistance if needed. Maximal walking speed (m/s) was measured for a 10-m distance [23]. The Berg Balance Scale (BBS) was used to assess balance by observing 14 different functional tasks [24]. The overall scores range from 0 (severely impaired) to 56 points (excellent). The timed up and go test (TUG) was used to assess balance and basic mobility skills [25]. Time was measured with a stopwatch, and the use of a walking aid was allowed in the TUG and maximal walking speed test. The participants performed the BBS barefoot and other tests using their regular shoes.

Comorbidity was defined using a modified version of the 18-item functional comorbidity index (FCI) [26] including data on the following conditions: (1) rheumatoid arthritis and other connective tissue diseases, (2) chronic asthma or chronic obstructive pulmonary disease, (3) Parkinson’s disease or multiple sclerosis, (4) osteoporosis, (5) coronary artery disease, (6) heart failure, (7) myocardial infarction, (8) stroke, (9) diabetes, (10) depression, (11) visual impairment, (12) hearing impairment and (13) obesity. Cognitive function was assessed using the Mini-Mental State Examination (MMSE) [27] and self-rated health was assessed with the following question: “How would you rate your health at the moment?” The participants selected one of five responses. In the analysis, three categories were used: (1) good or very good, (2) moderate and (3) poor or very poor.

The ability to perform instrumental activities of daily living (IADL) was assessed using the eight-item Lawton and Brody Instrumental Activities of Daily Living Scale, with ratings from 0 to 8, with higher scores indicating better functioning [28]. The level of physical activity was assessed using a modified version of the Grimby Scale [29]. The participants were categorized on the basis of their self-rated physical activity into low (no other exercise beyond light walking one to two times/week), moderate (light walking or other light exercise several times/week, or moderate exercise one to two times/week) or high (moderate or vigorous exercise several times/week) activity levels.

### SBT intervention

The participants had an opportunity to participate in group-based SBT, supervised by a physiotherapist, once a week between September 2004 and December 2006. Training was organized in small groups in the city center and was free of charge. Each training session started with a 15-min balancing exercise as a warm-up. This included different kinds of static and dynamic, standing, walking, turning and reaching exercises where challenge was adjusted by changing the size or stability of the base of support. Also dual task and eyes-closed situations were applied.

This was followed by 60 min of progressive resistance training which included knee extension and flexion, leg press, hip adduction, abduction and extension and abdominal crunches (Technogym SpA, Cesena, Italy). The intensity of strength training was determined individually by an indirect method to evaluate one repetition maximum (1 RM): after a couple of introductory training sessions, the prediction of training load was evaluated using 3–6 repetitions and the formula reported by McDonagh and Davies [30]. Based on this the load for training was set to be 60–85% of 1 RM.

Participants were instructed to perform 8–12 repetitions and two to three sets of the exercises. The resistance was adjusted throughout the intervention, and progression was accomplished by increasing the load while maintaining the same number of repetitions. Training adherence was measured by the number of training sessions attended relative to the number of training sessions offered, and expressed as adherence percentage. During the 2.3-year intervention period, the participants’ mean adherence to SBT was 55% (SD 29, range 1–99%); 57% (SD ± 28) for women and 49% (SD ± 28) for men (*p* = 0.07) [31].

### Statistical analysis

Descriptive statistics were expressed as the means with standard deviations (SD), medians with interquartile ranges [IQR] or as counts with percentages. The statistical significance of the difference between the men and women was analyzed with a *T* test, a Chi-square test or a Mann–Whitney *U* test when appropriate.

The effects of the training intervention were analyzed separately for women and men due to differences in baseline characteristics and performance level. A linear mixed model was used to examine the change in physical functioning over time in men and women. An unstructured covariance matrix was used to estimate the variance of the random intercepts. The mixed model approach used all the available data on each subject and was the method which best accounted for observations missing at random. Time effect within men and women was estimated with age, years of education, cognition, IADL, depressive symptoms and physical activity adjusted models. First, the analyses were carried out for the 2-year intervention, after which the 1-year follow-up was included in the analyses. An alpha level of *p* < 0.05 was set for the level of significance. SPSS for Windows version 20.0 (SPSS Inc., Chicago, Il, USA) was used to conduct the analyses.

## Results

Among women, the knee extension (*p* = 0.003), and knee flexion strength improved (*p* < 0.001), during the 2-year intervention (Table 2). Compared with the baseline level, both extension (*p* = 0.02) and flexion (*p* < 0.001) strengths were higher at the end of the subsequent follow-up. Men’s strength levels did not change during the training period or over the subsequent follow-up.

**Table 2:** Means and standard deviations (SD) of observed physical functioning outcomes, and linear mixed model estimates for change in physical functioning over 2-year intervention, and post-intervention by men and women

	Baseline	1 year	2 year	Estimated change from baseline to 2 years		Follow-up	Estimated change from baseline to 3 years	
	Mean (SD)	Mean (SD)	Mean (SD)	*β* (SE)	*p* value	Mean (SD)	*β* (SE)	*p* value
Knee extension strength (N)								
Women	268 (75)	286 (80)	291 (77)	14.1 (4.58)	*p* = 0.003	290 (76)	10.59 (4.47)	*p* = 0.02
Men	404 (108)	418 (122)	414 (113)	− 5.5 (7.51)	*p* = 0.47	408 (112)	− 7.4 (8.47)	*p* = 0.39
Knee flexion strength (N)								
Women	114 (39)	130 (39)	135 (42)	16.3 (2.86)	*p* < 0.001	135 (39)	12.3 (2.96)	*p* < 0.001
Men	200 (56)	203 (55)	204 (55)	− 0.38 (5.10)	*p* = 0.94	202 (62)	− 7.49 (5.39)	*p* = 0.17
Chair rise (s)								
Women	16.3 (5.7)	13.2 (4.5)	12.8 (4.5)	− 2.47 (0.53)	*p* < 0.001	11.8 (2.8)	− 2.66 (0.46)	*p* < 0.001
Men	14.4 (3.9)	13.6 (5.3)	12.8 (3.7)	− 1.44 (0.52)	*p* = 0.013	14.0 (5.8)	− 0.37 (0.81)	*p* = 0.65
Walking speed (m/s)								
Women	1.28 (0.33)	1.42 (0.32)	1.41 (0.35)	0.08 (0.02)	*p* < 0.001	1.45 (0.35)	0.07 (0.03)	*p* = 0.009
Men	1.49 (0.37)	1.53 (0.40)	1.51 (0.40)	− 0.02 (0.04)	*p* = 0.59	1.52 (0.44)	− 0.04 (0.05)	*p* = 0.37
Timed up and go (s)								
Women	11.0 (4.1)	9.8 (4.1)	10.0 (3.6)	− 0.21 (0.45)	*p* = 0.64	9.4 (2.8)	0.33 (0.51)	*p* = 0.52
Men	10.6 (5.5)	10.1 (5.2)	10.4 (5.8)	0.30 (0.48)	*p* = 0.58	11.1 (6.5)	1.51 (0.75)	*p* = 0.05
Berg Balance Scale (points)								
Women	51.4 (4.2)	52.4 (3.7)	52.4 (3.8)	0.45 (0.52)	*p* = 0.39	52.6 (3.3)	− 0.67 (0.60)	*p* = 0.27
Men	51.6 (5.1)	51.7 (5.4)	52.0 (4.8)	− 0.63 (0.52)	*p* = 0.73	51.2 (5.4)	− 2.43 (1.09)	*p* = 0.04

During the training period, both women (*p* < 0.001) and men (*p* = 0.013) improved their chair rise performance. For women the improved chair rise performance remained by the end of the follow-up, while for men the chair rise performance declined back at the baseline level. Women improved their maximal walking speed (*p* < 0.001) during the intervention and the improvement was maintained over the follow-up, while in men, maximal walking speed remained unchanged. TUG and BBS performance did not change from the baseline level during the intervention. However, by the end of the post-intervention follow-up, BBS declined (*p* = 0.04) for men.

## Discussion

The decline in older adults’ physical functioning appears not to be linear but to accelerate with increasing age. Physical exercise is currently the only intervention that has been shown to effectively improve muscle strength in old age [32] and resistance training being an integral component to prevent age-related decline in muscle strength [33]. Thus, the main findings of this long-term intervention study among a community sample of older adults aged 75–98 years are noteworthy. The findings of this study suggest that long-term strength and balance training once-weekly improves or maintains muscle strength and mobility in older adults. Both men and women improved their chair rise performance, and women improved their walking speed and knee flexion and extension strength. The changes achieved were partially maintained during the post-intervention follow-up, except for chair rise performance for men. These results are exploratory, as there was no control group. However, taking into account the mean participant age of 80 at baseline and the 2-year training period, the results are encouraging and support the implementation of SBT in older community populations to support their independent mobility.

In this study, a positive change on muscle strength was observed for women. Knee extension strength improved by 5.3%, and knee flexion strength improved by 14.3% during the intervention. The absolute average changes were small (~ 1.5 kg). At the same time, men’s strength levels maintained during the intervention, which may be seen as a positive training effect in this age group. The strength gains were partially maintained after the intervention ended. In women, both knee extension (4.0%) and flexion (10.8%) strengths were higher at the end of the post-intervention follow-up compared with baseline. A previous study with 60–80-year-old participants showed how the intervention group with 1-year extensive strength training had better muscle strength even 7 years after enrollment compared to the control group, but training did not attenuate the age-related decline in muscle strength [34]. It continued similarly in both groups once the training stopped. In our study, the post-intervention decline in muscle strengths did not show in men or women when compared to baseline.

The effects of long-term SBT among older adults with comorbidities and mean age of 80 years are scarce. Previous meta-analysis has presented a large positive effect of progressive resistance training on muscle strength in people 60 years and older [12]. The effect appears to also be positive on measures of physical functioning (i.e., balance, gait speed, TUG and chair rise) though the evidence is weaker than that on the muscle strength [12]. In our study, the greatest effects on physical functioning, although small, were found in the chair rise in both men and women and walking test in women. The training was centered on muscle strengthening in the lower extremities, and therefore it is consistent that the effect was greatest for these parameters [35, 36]. Relative to independent and safe mobility, this result is encouraging.

The TUG and BBS results remained at the baseline level during the intervention. In a previous systematic review, only weak evidence was found to support the theory that exercise is effective in improving balance outcomes [10]. This may be related to the “specific effect of training”. The present strength training occurred primarily in a sitting position, aside from the balance warm-up sessions. Therefore, it was possible that the training was not challenging enough for postural balance. Strength training as an isolated intervention has not been shown to be uniformly effective in improving balance performance [13]. Previously, in a short-term intervention, strength training alone improved walking speed, but not had any effect on standing balance or chair rise performance in active, community-dwelling older adults [37]. Another point is that while in the present long intervention period it was possible to make the strength training component progressive and more challenging, progression was less clear in the balance training. One reason for this is that methods for quantifying the level of the challenge to the individual’s balance system are lacking [38]. There is also a probable ceiling effect with the used outcome measure, with the participants’ BBS scores already high at baseline, thus preventing significant improvement. However, the BBS test showed statistically significant declines during the post-intervention follow-up for men.

The baseline performance in strength and timed functional tests is not comparable between men and women. However, women used walking aids more often. This suggests that women had higher frequencies of mobility limitation and were on a lower functional level than men. Based on our results women benefited more than men from the weekly SBT. This is consistent with the findings of a previous study according to which those with lower initial strength and function benefited more from training [39]. The men had good average maximal walking speed (1.5 m/s) at baseline and large muscle strength gains would have been needed to induce a notable increase in walking speed [36].

During the long-term intervention, the mean adherence of 55% indicates that the average training frequency was once every 2 weeks. Although the American College of Sports Medicine has previously recommended strength training be done two times per week for adults aged 65 and over [40], there is some evidence to support the idea that training once per week might be effective in increasing strength and preventing sarcopenia among older adults [19, 41]. Similarly, the recently updated physical activity guidelines for Americans state that older adults benefit from multicomponent training, even if they do not meet the key guidelines [7]. Despite the relatively low training frequency in this study, the intensity was planned to be on a level (60–85% 1RM) conforming with the previous recommendations [40]. Lower limb strength will improve more by higher training intensities (70–89% of 1RM), while interestingly, the functional performance might improve by moderate (50–69% of 1RM) or even low (≤ 50% of 1RM) training intensities [42]. However, the dose–response relationship between training intensity and gains in strength and physical functioning shows that high intensity training would be more effective than low intensity training [43]. Taken together, for very old and frail populations, the effect of different training volumes and frequencies, as well as the dose–response relationship, still needs to be confirmed [16, 44].

The strengths of the current study are the community-based setting and the considerably long-term training intervention. As few exclusion criteria as possible were set, so that the sample also included oldest-old individuals with several comorbidities, thereby reflecting real-world situations and improving generalizability of the results among community-dwelling older populations. In this study, the training was preceded by a comprehensive geriatric assessment and a screening for contraindications. Objective measures of functional status were used annually as part of the comprehensive geriatric assessment conducted by health care professionals. The training was professionally supervised, and the progression was individually adjusted. No serious adverse events occurred during the intervention, although there were breaks from training due to hospital admissions and other personal reasons. This confirms the previous findings that high intensity strength training is feasible and safe for older adults.

### Study limitations

Our study clearly has some limitations. Our study was not a single intervention study but included also other health promoting interventions, such as optimizing medication and nutrition. However, this multi-intervention promoted the safe implementation of long-term strength training in a heterogeneous population sample. It was also possible that participants continued training independently or in other supervised groups after the SBT intervention, and this may have had influenced the post-intervention follow-up measurements. The fact that inter-rater reliability for outcome measures was not assessed can be regarded as a limitation of this study. However, trained physiotherapists conducted the measurements and each participant was annually assessed by the same therapist. These outcome measures have shown good reliability in previous studies when used by trained assessors.

### Practical applications

Once-weekly strength and balance training implemented in this study had a positive effect, especially on muscle strength and strength-demanding activities such as walking speed and chair rise in a community-dwelling population aged 75 years and older showing wide range of health and physical functioning. In physical activity counseling, it is also important to accept smaller increments in physical activity to potentially reap the benefit of maintaining one’s independent mobility. Even once-weekly training may represent a substantial increase in weekly physical activity and could reasonably be expected to help in the prevention of age-related loss of muscle strength and mass.

## Conclusions

In community-dwelling older adults with variety in health and functioning supervised strength and balance training once-weekly may help to prevent age-related decline in mobility and muscle strength when continued over 2 years. Even small increments in SBT could benefit in maintaining older adults independent mobility.
